# Providing curb availability information to delivery drivers reduces cruising for parking

**DOI:** 10.1038/s41598-022-23987-z

**Published:** 2022-11-11

**Authors:** Giacomo Dalla Chiara, Klaas Fiete Krutein, Andisheh Ranjbari, Anne Goodchild

**Affiliations:** 1 University of Washington, Civil & Environmental Engineering, Seattle, WA USA; 2 University of Washington, Industrial & Systems Engineering, Seattle, WA USA; 3 Pennsylvania State University, Civil and Environmental Engineering, State College, PA USA

**Keywords:** Civil engineering, Statistics

## Abstract

Delivery vehicle drivers are experiencing increasing challenges in finding available curb space to park in urban areas, which increases instances of cruising for parking and parking in unauthorized spaces. Policies traditionally used to reduce cruising for parking for passenger vehicles, such as parking fees and congestion pricing, are not effective at changing delivery drivers’ travel and parking behaviors. Intelligent parking systems that use real-time curb availability information to better route and park vehicles can reduce cruising for parking, but they have never been tested for delivery vehicle drivers. The current study tested whether providing real-time curb availability information to delivery drivers reduces the travel time and distance spent cruising for parking. A curb parking information system deployed in a study area in Seattle, Wash., displayed real-time curb availabilities on a mobile app called OpenPark. A controlled experiment assigned drivers’ deliveries in the study area with and without access to OpenPark. The data collected showed that when curb availability information was provided to drivers, their cruising for parking time significantly decreased by 27.9 percent, and their cruising distance decreased by 12.4 percent. These results demonstrate the potential for implementing intelligent parking systems to improve the efficiency of urban logistics systems.

## Introduction

In the past years, the world has experienced unprecedented growth in demand for last-mile deliveries. With more people having access to the internet and living in cities, online shopping has become a common habit. The range of goods that consumers buy online and receive at home has also grown to include products such as fresh and frozen food and prepared meals, which require even shorter delivery times. All of these have led to a growth in the number of delivery vehicles operating in cities^1^. This increase in the urban commercial vehicle fleet has not been met by a change in the urban infrastructure to support it^2^. Buildings in urban centers are often not equipped with suitable off-street loading/unloading bays, and carriers mostly rely on curb space to park and load/unload^3^. However, urban curb space is not only a limited resource, but it is increasingly becoming a multi-use domain: ridehailing vehicles, public transit vehicles, bicyclists, pedestrians, and commercial establishments, among others, claim use of the curb space^4^. Consequently, delivery drivers compete among each other and with other users for limited urban curb^5^. Moreover, urban planners have been re-allocating streets and curbs to pedestrians and alternative modes of transportation while reducing delivery vehicle access to central areas^6^. The last mile problem is increasingly becoming the last 50 feet problem—when a delivery driver pulls over, parks, and walks to a delivery destination^7^.

When demand for curb parking exceeds the supply, some drivers are not able to find available parking and therefore travel to curb spaces farther from their destinations^8^. This phenomenon has been termed cruising for parking^9^. Studies have estimated that vehicles cruise for parking between 3.5 and 13.9 min, and the share of traffic cruising for parking ranges between 8 and 74 percent of total traffic in urban areas^9^. However, these studies have focused only on passenger vehicle drivers.

Until recently, the paradigm in the scientific community was that commercial vehicles do not cruise for parking but instead double park. This paradigm has been recently challenged by two studies^10,11^ that showed that parking behaviors of commercial vehicle drivers are far more complex. A parcel delivery driver spends on average 2.3 min per trip and 1.1 h a day cruising for parking^10^. This means that when delivery drivers do not find an authorized parking space, not only do they double park, but they also park in unauthorized curb spaces, competing for curb space with other users. Following these research findings, this study explored how delivery drivers’ cruising for parking can be reduced by using information technology.

The traditional approach to reducing cruising for parking and cruising traffic has been to implement road and parking pricing policies. By increasing the cost to access and park in urban centers, cities incentivize travelers to change travel modes, departure times, trip destinations, or forgo their trips^12^. This would result in a decrease in demand and cruising for parking. However, pricing policies are less effective at influencing commercial vehicle travel behavior^13^. The ability of delivery drivers to change travel behavior is constrained by other participants in the supply chain, including receivers and shippers. Delivery trips are a form of “compulsory trips,” for which drivers do not make choices about travel time and mode.

### Intelligent parking systems

A different approach to managing curb parking is through intelligent parking systems^14^ (IPS). If cruising for parking is caused by a lack of information about curb availability, then by providing this information, cities can help drivers make better routing and parking decisions.

**Figure 1:**
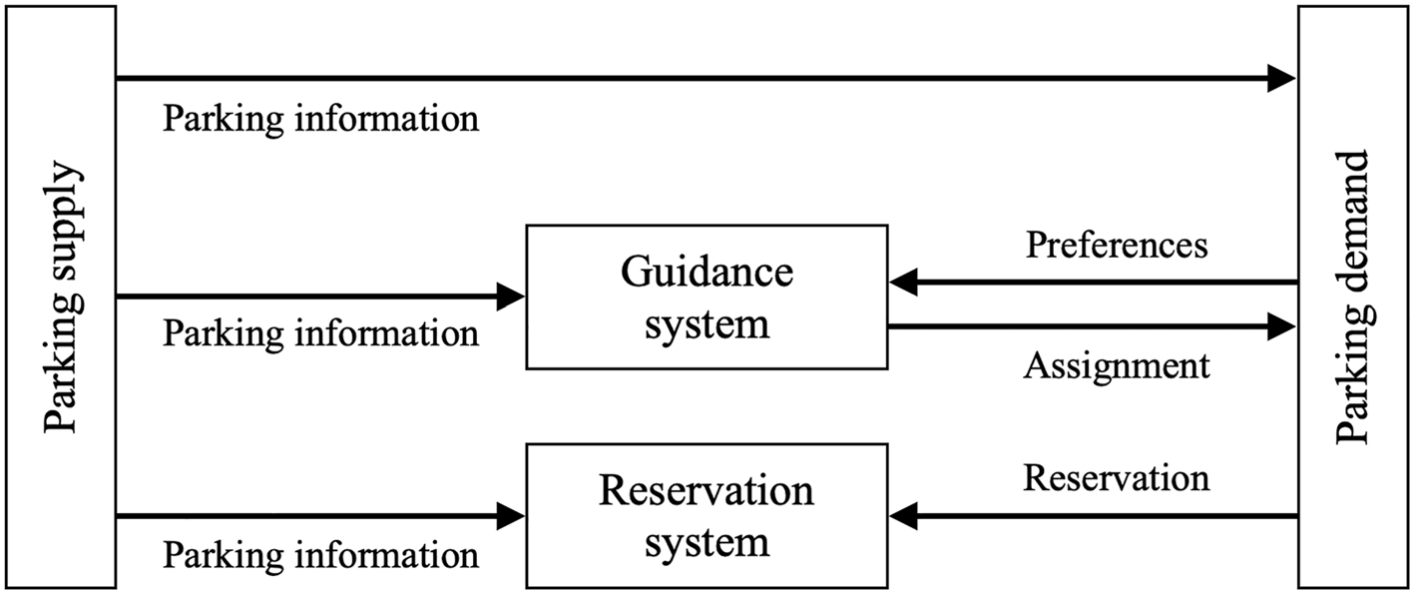
Typologies of intelligent parking systems.

There are different ways to communicate parking information to drivers, as shown in Fig. 1. Several studies have envisioned a centralized system ( “guidance system”) that takes as inputs curb availabilities on the one hand and travel destinations and parking preferences on the other hand, and then computes an optimal parking assignment^15–20^.

Another IPS model is a “reservation system,” in which a driver directly books a parking space for a given time window. Most papers have evaluated IPS from the perspective of passenger vehicles, and those that have considered IPS for commercial vehicles have focused on parking reservation systems^21–23^. McLeod and Cherrett^21^ were the first to apply the concept of an advanced booking system to loading/unloading bays. Roca-Riu et al.^22^ developed a method to solve the parking slot assignment problem for loading/unloading bays, while Mor et al.^23^ analyzed a booking system for loading/unloading areas. Several cities, such as Washington, D.C,. and Columbus, Ohio, have tested reservation systems for delivery vehicles^24^. However, these systems have been generally difficult to implement, as they depend on effective parking enforcement and users’ compliance^24^.

A simpler IPS informs drivers of curb availabilities and lets them decide how to use this information. Curb availability information can be provided through different media, including radio, variable message signs (VMS), and mobile applications. Previous studies have quantified the effect of providing drivers curb availability information^25–28^. However, these studies have focused only on passenger vehicle drivers and have mostly relied on data from simulation models or surveys. The current study is the first to test a curb parking information system for delivery drivers using data from a real-world experiment.

Recent technological advances and wider penetration of smartphones have made feasible the implementation of parking information systems. One such implementation was SFpark in San Francisco, Calif., where in-ground sensors were used to estimate occupancies and dynamically change parking prices to maintain occupancy levels between 60 and 80 percent^29^. Another parking information system called OpenPark was deployed in Seattle, Wash., where in-ground sensors provided real-time curb availability to delivery drivers through a mobile application. The OpenPark pilot was the subject of the study presented in this paper, and it represents the first parking information system targeted for commercial delivery drivers.

### OpenPark: a real-time curb availability information system

**Figure 2:**
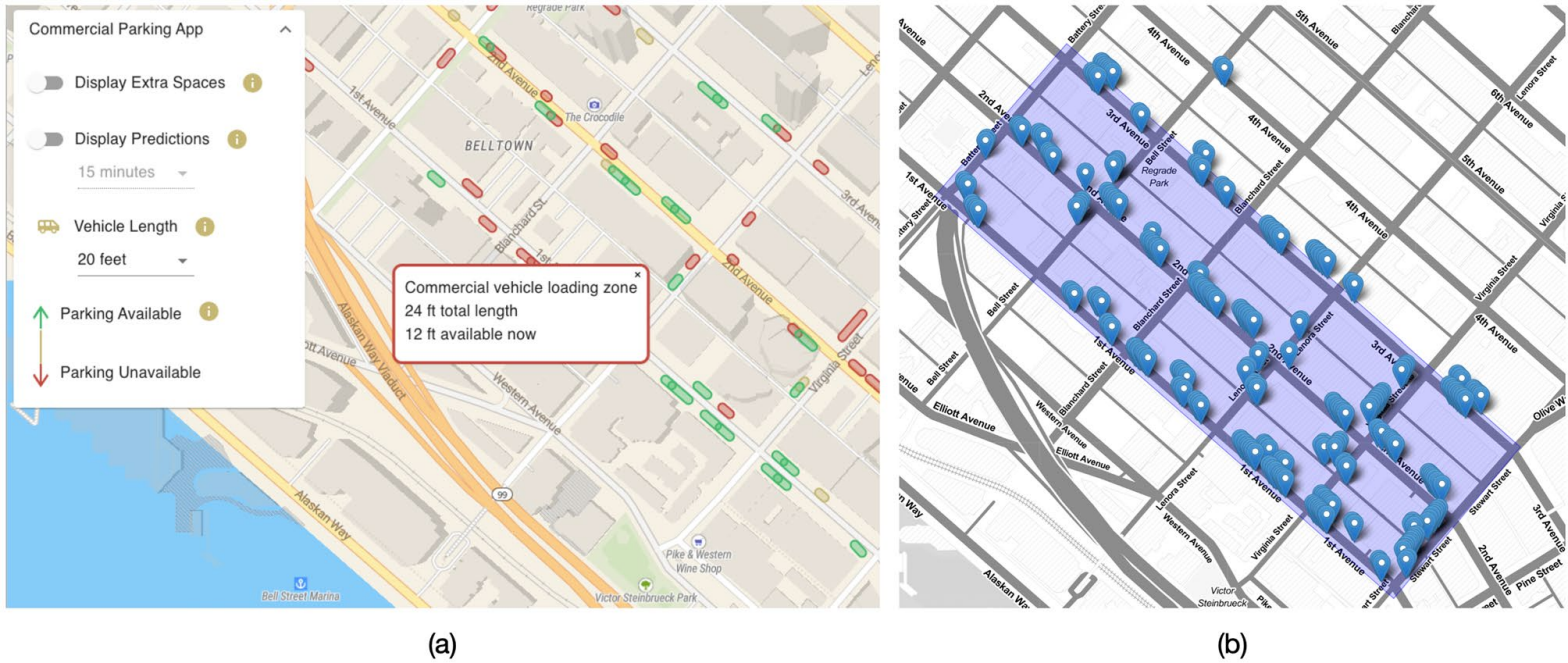
(**a**) OpenPark, a real-time curb availability information application. (**b**) Study area with curb sensor locations. The maps were created using Leaflet library^31^.

**Figure 3:**
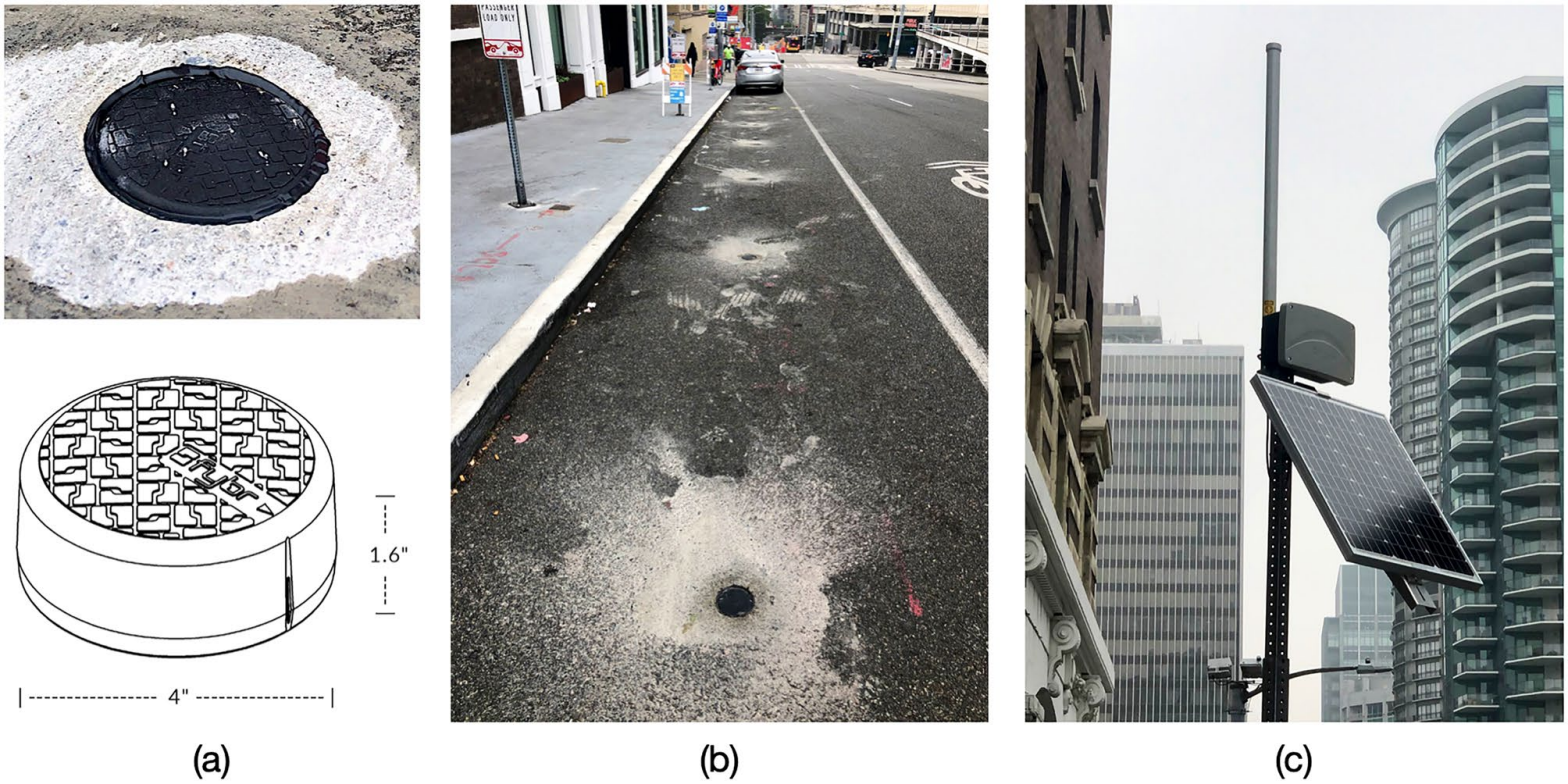
(**a**) Proximity sensors installed in the curb. (**b**) Curb sensors deployment in curb parking spaces. (c) Gateway transmitting and receiving sensors’ data.

OpenPark is a web-based platform (Fig. 2a) that displays real-time curb availabilities for a 10-block study area in the Belltown neighborhood of Seattle, Wash. (Fig. 2b). To gather real-time curb availability data, 274 in-ground proximity sensors were deployed in all commercial vehicle load zones (CVLZs), where vehicles with a commercial permit can park for up to 30 min, and all passenger load zones (PLZs), where vehicles can park for up to 3 min. Each sensor used a magnetometer to detect electromagnetic perturbations generated by vehicles^30^ (Fig. 3a). Sensors were placed 10 feet apart and 5 feet from the beginning/end of each curb zone (Fig. 3b), and they transmitted occupancy data in real time wirelessly through a network of communication equipment (Fig. 3c).

### Research objective

The objective of the current study was to estimate the impact of providing curb availability information on delivery drivers’ cruising for parking behavior. Understanding how delivery drivers behave in the presence of curb availability information will help cities and carriers determine how and to what extent these systems could improve the efficiency of the urban logistics system.

In this work, delivery drivers’ parking behaviors were experimentally analyzed in a real-world scenario. In a study area located in Seattle, Wash., in-ground proximity sensors were installed in curb spaces. Sensors’ data were used to display real-time curb availabilities on the OpenPark mobile application (shown in Fig. 2a). A real-world controlled experiment was designed in which drivers were assigned delivery tasks within the study area, with some delivery tasks performed with OpenPark and others performed without. GPS data were collected during the experiment, and the study assessed the impact of providing delivery drivers with real-time curb availability information on both their cruising for parking time and distance and the total vehicle travel time and distance.

## Methods

### Experimental design

To test whether curb availability information affects delivery drivers’ cruising for parking behavior, a real-world experiment was conducted. Drivers were hired to perform mock deliveries in the study area. Each driver was provided with a 20-foot delivery van and three delivery manifests—lists of delivery tasks to be performed. Each manifest contained a pre-generated list of 15 addresses randomly sampled from the addresses in the study area, to be visited within a single route, i.e., starting from a parking lot located near the study area (the “depot”) and returning to it once all delivery tasks had been completed. A mock package was placed in the trunk of the delivery vehicle for the driver to carry to simulate a functional delivery.

At the start of a route, each driver received a tablet displaying a map of the study area and the locations of the delivery addresses. On some of the routes, drivers were also provided with the OpenPark app, displaying real-time curb availability information. Upon opening the app, drivers selected their vehicle length. Then available curb spaces were shown in green, unavailable spaces in red, and spaces that are available but not long enough for their vehicle were shown in yellow. Changes in curb availability were reflected in the app with a latency of 5 s. Drivers were allowed to access the tablet only when parked.

A driver would then start the route by driving to the vicinity of a delivery address, parking the vehicle, and walking to the entrance of the building. Drivers were free to choose:

- how many times and where to stop the vehicle,
- to which delivery address(es) to walk from each stop and
- in which order to perform the deliveries.

Eleven drivers were hired and ten different delivery manifests were generated. Both experienced drivers (currently employed by delivery carriers) and non-experienced drivers (students with some experience as delivery drivers but not currently employed) were recruited. The sequence of assigned delivery manifests and whether a driver started with a delivery manifest using the OpenPark app were randomized.

Manifests were allocated to drivers such that each manifest was performed at least once with the app and once without (see Table 1). Each driver followed three different manifests, at least once with the app and once without. Drivers also followed a shorter, five-delivery manifest at the beginning of the day to gain understanding of the study area and the required tasks. Data from the mock manifests were not used in the analysis.

**Table 1:** Experimental design.

Drivers	Manifests										Total no. routes
	M1	M2	M3	M4	M5	M6	M7	M8	M9	M10	
**D1**	No app	No app							App		3
**D2**		App	App	No app							3
**D3**			App		No app	No app					3
**D4**	App					App	No app				3
**D5**		No app		No app				App			3
**D6**				No app	App		No app				3
**D7**						App	No app	App			3
**D8**			No app		App				No app		3
**D9**	No app			App			App				3
**D10**				App				No app		No app	3
**D11**							App	No app		App	3
**Total no. routes**	3	3	3	5	3	3	5	4	2	2	33

No app, route was performed without access to OpenPark app; App, route was performed with access to OpenPark app.

### Data collection

The controlled experiment was conducted on weekdays between July and November 2021, between 8:00 am and 2:00 pm. Drivers performed 33 routes (11 drivers, each carrying out three different delivery manifests a day from a set of ten manifests), completing 495 deliveries. Drivers made 177 trips to reach the delivery destinations (more than one delivery could be made from a single stop). Of the total, 91 trips and 17 routes (51 percent) were performed without OpenPark, and 86 trips and 16 routes (49 percent) were performed with OpenPark.

Drivers were provided with the same delivery van and performed the assigned manifests starting at 9:00 am on a weekday from the depot and completing all three routes by 1:00 pm. They were also instructed to not double park (park in the travel lane) to avoid potential safety concerns. However, the drivers were still free to choose where to park the vehicle.

An observer followed each driver, collecting GPS traces and distinguishing between in-vehicle segments (traces collected while driving) and out-of-vehicle segments (traces collected while walking to/from a delivery address). For each in-vehicle segment ending in a parking stop, the trip start/end times, the total trip duration, and trip distance were recorded. The following four main variables of interest were then computed:

- *Trip time*: time in minutes it took to reach a parking stop location.
- *Trip distance*: km traveled to reach a parking stop location.
- *Route travel time*: total time the driver spent in the vehicle, computed by summing all trip times in a route.
- *Route travel distance*: total km traveled to perform a route.

### Modeling cruising for parking

To understand the impact of curb availability information on delivery drivers’ cruising for parking behaviors, a mixed-effect random intercept model^32^ was used. This model accounted for possible dependencies between observations generated by the same driver and delivery manifest^32^. Four models, one for each variable of interest, were estimated by using the data obtained in the controlled experiment.

The model for trip time is shown in Eq. (1). Consider driver $i$, performing delivery manifest $j$, driving the vehicle to stop location $k$. The total trip time is ${TripTime}_{ijk}$, measured in minutes. The logarithm transformation of trip time is regressed over the following:

- the expected driving time ${DriveTime}_{ijk}$ if the driver had perfect information on available parking i.e., the time it takes to reach a parking destination without having to cruise for parking;
- an indicator variable $1\left[{App}_{ijk}\right]$ with a value $=1$ whenever the OpenPark app displaying real-time curb availability information was provided to the driver, and 0 otherwise;
- two vectors of indicator variables, $\varvec1\left[{Day}_{ijk}\right]$, and $\varvec1\left[{Hour}_{ijk}\right]$, which controlled for the day of the week and the time of day.

Parameters ${{\beta }_{0},{\beta }_{1},\beta }_{2}$, ${{\varvec{\gamma}}}^{\prime}$ and ${{\varvec{\delta}}}^{\prime}$ are the regression fixed effects, ${u}_{i}$ and ${u}_{j}$ are the random intercepts for driver $i$ and manifest $j$, and ${\varepsilon }_{ijk}$ is the zero-mean Gaussian error term. The logarithm transformation was used not only for ease of coefficient interpretation, but also because the dependent variable is strictly positive, and therefore its conditional distribution might be skewed or heteroskedastic, which might affect model inference^33^.

1 $$\begin{aligned}\mathrm{log}({TripTime}_{ijk})& = \left({\beta }_{0}+{u}_{i}+{u}_{j}\right)+{\beta }_{1}\mathrm{log}({DriveTime}_{ijk})+{\beta }_{2}1\left[{App}_{ijk}\right]\\&\quad+{{\varvec{\gamma}}}^{{^{\prime}}}\varvec1\left[{Day}_{ijk}\right]+{{\varvec{\delta}}}^{{^{\prime}}}\varvec1\left[{Hour}_{ijk}\right]+{\varepsilon }_{ijk}\end{aligned}$$

The coefficient of interest is ${\beta }_{2}$, measuring the effect of exposure to real-time curb availability information on the total trip time ${TripTime}_{ijk}$, controlling for the driving time ${DriveTime}_{ijk}$, and the other regressors. Coefficient ${\beta }_{2}$ can be interpreted as the effect of providing curb availability information on cruising for parking, alternatively stated as the additional time required to find parking because the availability of parking was unknown.

A formula like Eq. (1) was used to model trip distances, shown in Eq. (2). The logarithm of trip distance was used as the dependent variable, and the expected travel distance was used as an independent variable, while all other variables remained the same as in Eq. (1).

2 $$\begin{aligned}\mathrm{log}({TripDist}_{ijk}) &= \left({\beta }_{0}+{u}_{i}+{u}_{j}\right)+{\beta }_{1}\mathrm{log}({DriveDist}_{ijk})+{\beta }_{2}1\left[{App}_{ijk}\right]\\&\quad+{{\varvec{\gamma}}}^{{^{\prime}}}\varvec1\left[{Day}_{ijk}\right]+{{\varvec{\delta}}}^{{^{\prime}}}\varvec1\left[{Hour}_{ijk}\right]+{\varepsilon }_{ijk}\end{aligned}$$

**Figure 4:**
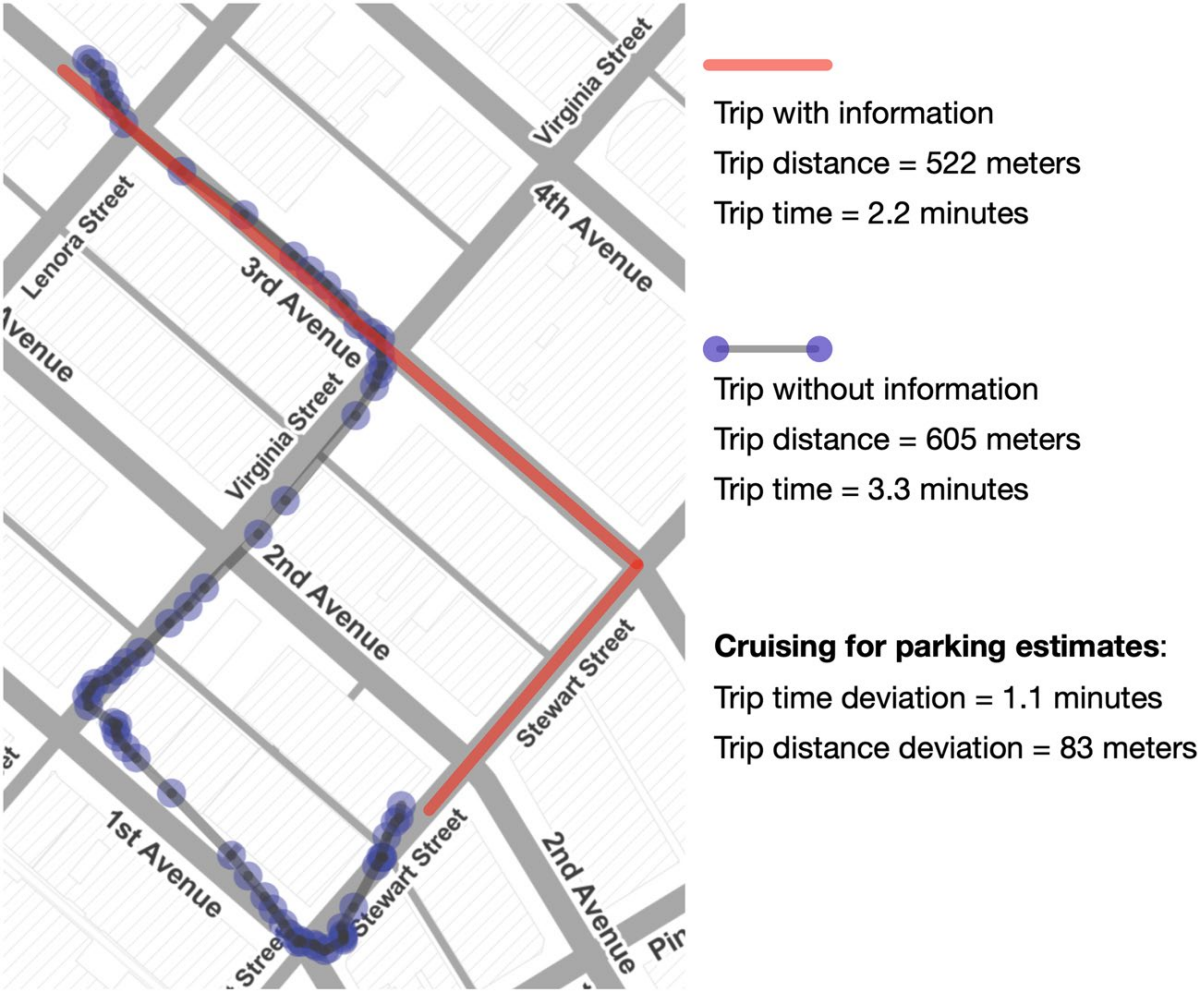
Estimation of cruising for parking time and distance. The map was created using the Leaflet library^31^.

Trips times and distances were obtained from the GPS data collected. Following the methodology developed by Dalla Chiara and Goodchild^10^, expected driving times/distances were estimated by querying the Google Maps Distance Matrix API^34^. Google Maps has been used as a reliable source of driving time estimates in previous studies^10,35–37^. It takes as inputs a trip origin, destination, departure time, day of the week, and travel mode, and it returns the expected driving time given historical road traffic data. While this estimate takes into consideration road traffic conditions, it does not consider curb parking occupancies; hence it doesn’t estimate the additional time spent cruising for parking. Therefore, we considered the difference between the real trip time ${TripTime}_{ijk}$ and driving time ${DriveTime}_{ijk}$ to be an estimate of cruising for parking time, and the difference between real trip distance ${TripDist}_{ijk}$ and the expected driving distance ${DriveDist}_{ijk}$ to be an estimate of cruising for parking distance. Figure 4 shows an example of a real trip time and distance, and their respective drive time and distance estimated from Google Maps.

Finally, total route travel time and distance were defined as the sum of the trip times and distances that a route comprised and were also modeled as mixed-effect random intercepts regressions, where the dependent variables were the total route time and route distance, and the independent variables were the indicator variable for access to OpenPark app and the indicator variables controlling for time of day and day of the week (Eqs. 3 and 4).

3 $$\mathrm{log}({RouteTime}_{ijk}) = \left({\beta }_{0}+{u}_{i}+{u}_{j}\right)+{\beta }_{2}1\left[{App}_{ijk}\right]+ {{\varvec{\gamma}}}^{{\prime}}\varvec1\left[{Day}_{ijk}\right]+{{\varvec{\delta}}}^{\prime}\varvec1\left[{Hour}_{ijk}\right]+{\varepsilon }_{ijk}$$

4 $$\mathrm{log}({RouteDist}_{ijk}) = \left({\beta }_{0}+{u}_{i}+{u}_{j}\right)+{\beta }_{2}1\left[{App}_{ijk}\right]+{{\varvec{\gamma}}}^{\prime}\varvec1\left[{Day}_{ijk}\right]+{{\varvec{\delta}}}^{\prime}\varvec1\left[{Hour}_{ijk}\right]+{\varepsilon }_{ijk}$$

Using the sample of 177 trip times and distances and 33 route times and distances collected during the experiment, the regression coefficients for Eqs. (1–4) were estimated by Restricted Maximum Likelihood (REML), using the Lme4 package^32^, coded in the R programming language^38^.

### Ethics declarations

The University of Washington Human Subject Division (HSD)—Institutional Review Boards (IRBs) committee B determined that the current research is exempt from the IRBs review requirement (IRB ID: MOD00004331). The study used de-identifiable data and written informed consent was received from all participants in the study. All experiments and methods were performed in accordance with relevant guidelines and regulations.

## Results

### Descriptive results

**Figure 5:**
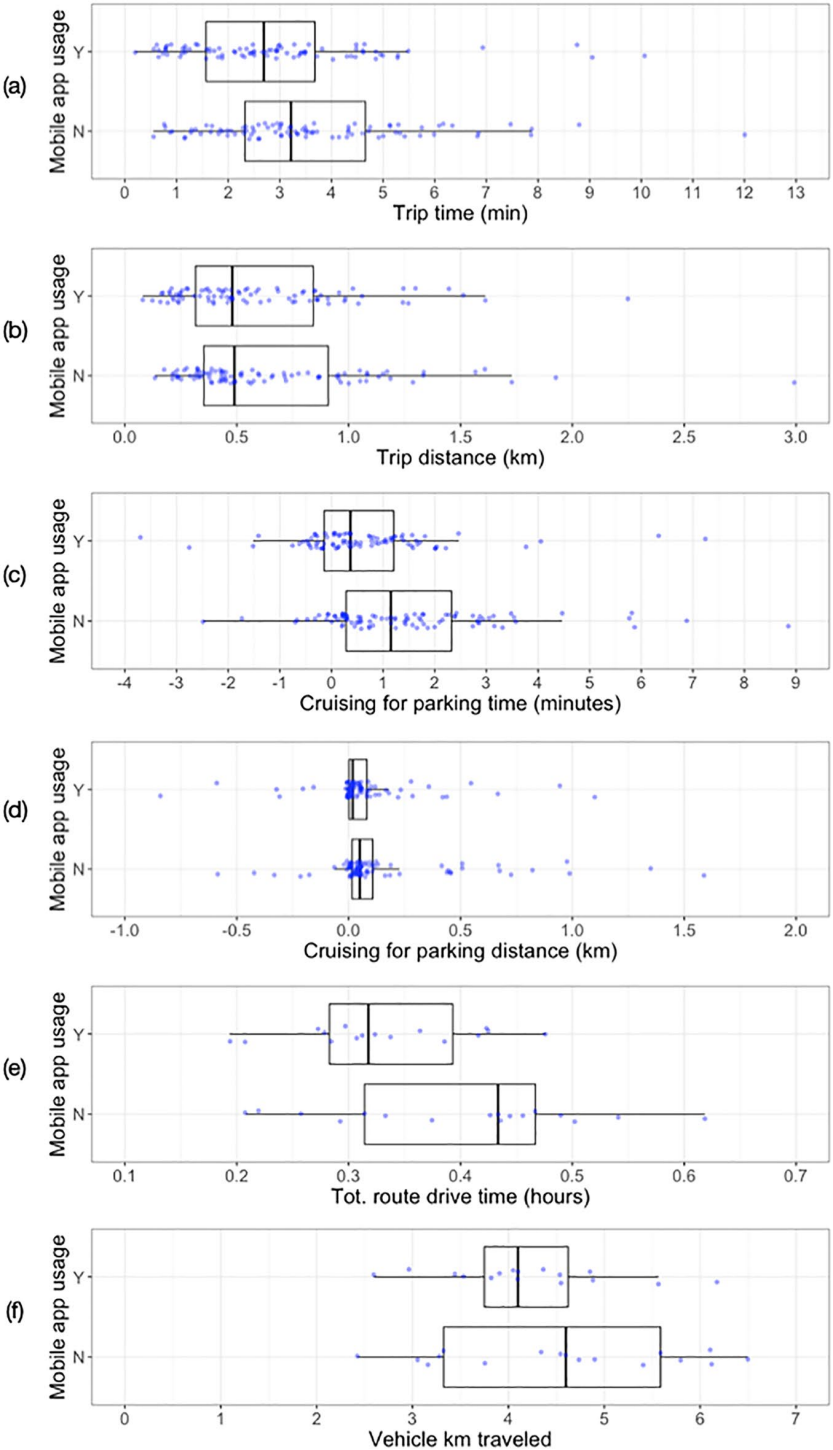
Empirical distributions of the variables of interest collected by performing routes with (mobile app usage = Y) and without (mobile app usage = N) the mobile parking information application.

Figure 5 shows, for each variable of interest, the empirical distribution obtained from trips for which drivers were provided curb availability information (app usage = Y) and trips for which drivers were not provided that information (app usage = N). The descriptive statistics of each distribution are reported in Table 2.

**Table 2:** Descriptive statistics of variables of interest.

Variable		Use app	Descriptive statistics					
			Min^a^	Med^b^	Mean	Max^c^	SD^d^	%$\Delta$ Med^e^ (%)
a	Trip time (minutes)	N	0.55	3.22	3.61	12.00	1.99	− 16.46
		Y	0.20	2.69	2.90	10.07	1.88	
b	Trip distance (km)	N	0.13	0.49	0.66	2.99	0.46	− 2.04
		Y	0.08	0.48	0.60	2.25	0.39	
c	Cruise time (minutes)	N	− 2.48	1.15	1.49	8.85	1.78	− 67.82
		Y	− 3.70	0.37	0.66	7.25	1.47	
d	Cruise distance (km)	N	− 1.74	0.05	0.15	2.39	0.44	− 60.00
		Y	− 2.56	0.02	0.01	1.10	0.42	
e	Total route drive time (hours)	N	0.21	0.43	0.40	0.62	0.12	− 34.38
		Y	0.19	0.32	0.33	0.48	0.08	
f	Total route distance (km)	N	2.42	4.60	4.57	6.50	1.24	− 6.92
		Y	2.60	4.10	4.21	6.18	0.91	

^a^Minimum;^b^median;^c^maximum;^d^standard deviation;^e^percentage change in median.

**Table 3:** Regression models estimation results.

Statistics	Models			
	Cruise time	Cruise distance	Route drive time	Route drive distance
Dependent variable	Trip time	Trip distance	Route time	Route distance
**App usage effect**				
Estimate^*a*^	− 0.327	− 0.132	− 0.174	− 0.044
Standard Error	0.079	0.072	0.091	0.084
P-value^b^	< 0.001	0.059	0.043	0.561
95% confidence interval^c^	(− 0.488, − 0.190)	(− 0.269, 0.005)	(− 0.340, − 0.006)	(− 0.203, 0.113)
**Random effects**				
${\sigma }_{driver}$	0.051	0.000	0.000	0.064
${\sigma }_{manifest}$	0.046	0.010	0.087	0.053
${\sigma }_{residual}$	0.045	0.419	0.251	0.232
**Summary statistics**				
Data source	Trips	Trips	Routes	Routes
Sample size	177	177	33	33
Log Likelihood	-105.6	− 90.5	0.4	3.1
AIC	243.2	211.0	17.1	11.9
BIC	294.1	258.6	30.6	25.3

^a^Estimates of regression coefficient ${\beta }_{2}$ in Eqs. (1–4) above.

^b^Computed by Likelihood Ratio Test.

^c^Computed by profiling the likelihood of the models using the *profile()* function in lme4 package^32^.

The median trip time decreased by 0.5 min (a 16.5 percent decrease) when the drivers were provided with curb availability information (Fig. 5a). The trip distances decreased by 10 m, a 2 percent decrease (Fig. 5b). In Fig. 5c, d the median cruising time and distance both decreased by 68 and 60 percent, respectively, when drivers were given information. Therefore, when drivers were provided with OpenPark, their cruising time was on average about 50 s and 30 m shorter.

When looking at the results from the route perspective, we observe a 6.6-min decrease in total route time spent driving (Fig. 5e) and a 500-m decrease in total route distance (Fig. 5f), which respectively correspond to a 27 and 11 percent decrease in comparison to routes completed without curb availability information.

### Modeling results

To analyze the impact of providing real-time curb availability information on drivers’ cruising for parking behavior, four regression models were estimated with trip time, trip distance, route time, and route distance as dependent variables. Each model had among its regressors an indicator variable with a value of 1 if the drivers received information and 0 otherwise. All the regression models considered possible dependencies between observations generated by performing the same manifest and with the same driver, adding driver and manifest random effects. The estimates for the coefficient of the indicator variable for the OpenPark usage for the four models are reported in Table 3, together with estimates of the parameters associated with the random effects and summary statistics describing the models’ fit.

All estimated coefficients for the effect of providing curb availability information on the variables of interest were negative, reflecting a trend similar to that of the descriptive results, that providing information reduced both cruising for parking time and distances and, consequently, the total route time and distance. The largest magnitude was observed in the effect on cruising for parking time, followed by route drive time, cruising for parking distance, and route drive distance. We also observed that providing curb availability information had a statistically significant (p-value < 0.05) impact only on cruising for parking time and route drive time. Information provision showed a less statistically significant impact on cruising for parking distance, and its impact on route driving distance was not significantly different from zero.

**Figure 6:**
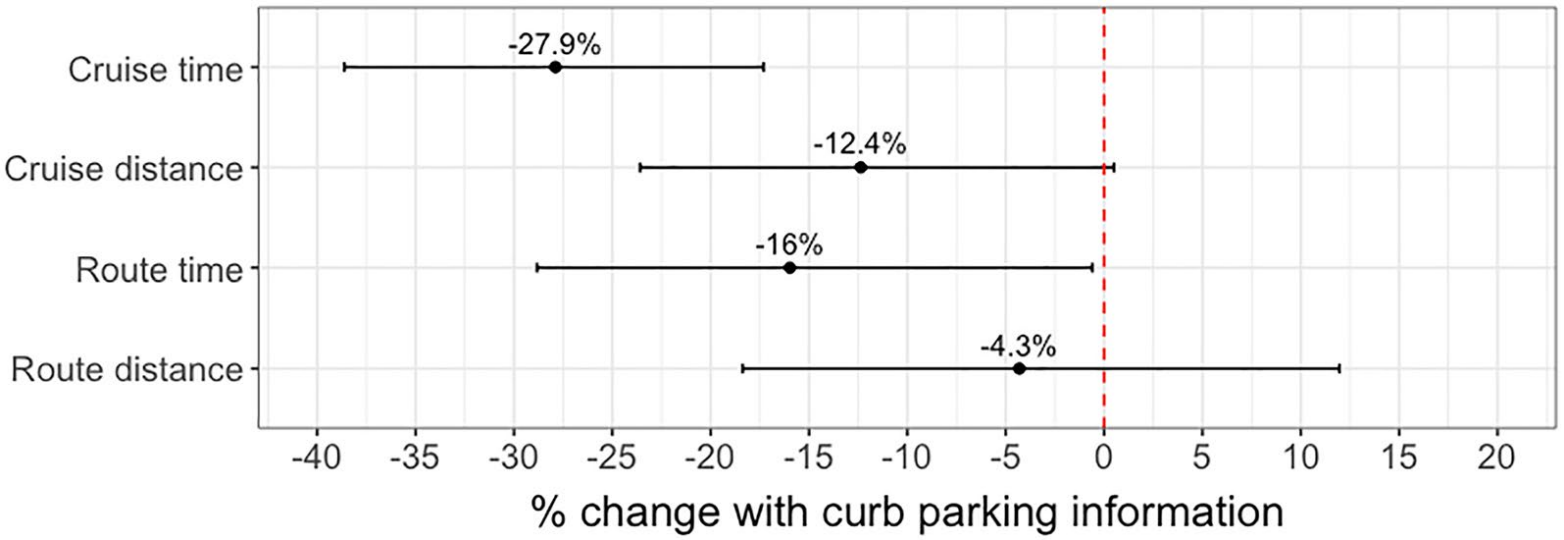
Estimated impact and 95 percent confidence interval of providing delivery drivers with curb availability information on the main variables of interest.

The above results can be interpreted as follows (see Fig. 6). When drivers were provided with curb availability information, their cruising for parking time was 28 percent shorter (obtained through $100[{e}^{-0.327}-1]$) than that of uninformed drivers. This improvement in efficiency was also reflected in the total route time spent driving, although by a smaller magnitude; total route time spent driving decreased by 16 percent when OpenPark was used. However, the impact of providing curb availability information on cruising for parking distance and total route travel distance was of a smaller magnitude. While information provision still had an impact on cruising for parking distance, with an estimated reduction of 12 percent, the impact of total route travel time was not significantly different from zero (with an estimated coefficient of -4.3 percent).

## Discussion

While modern navigation systems have seen huge developments in the past decade, drastically changing the way vehicles navigate through cities, parking information systems are still behind. The task of finding parking is still left to drivers and largely unsupported by technology. The operational inefficiencies generated by a lack of available parking for the urban logistics system are hefty, with delivery drivers parking in unauthorized spaces and spending hours a day cruising for parking.

The current work addressed these challenges with the goal of supporting delivery drivers’ parking decision-making. We showed that providing curb availability information significantly changes delivery drivers’ parking and routing behaviors. In a real-world controlled experiment, drivers were able to reduce the time spent cruising for parking by 28 percent and the total time spent driving on a tour by 16 percent when curb availability information was provided. A smaller magnitude impact was observed on cruising for parking distance (− 12.4 percent) and total route distance, (− 4.3 percent).

These estimates were in line with qualitative observations obtained by following the drivers. We observed a change in decision-making behavior when drivers had access to curb availability information: instead of choosing a block at which to start cruising, drivers targeted specific curb spaces that were readily available and directly drove there. Consequently, the certainty of the availability of the chosen curb space reduced the time drivers had to spend searching for parking, therefore reducing the cruising for parking time and, to some extent, also the cruising for parking distance, compared to un-informed drivers. The smaller magnitude of the impact of curb availability information on cruising for parking distance is explained by considering the fact that cruising for parking does not always involved driving longer distances (e.g. by circling the block to search for an available curb space), instead, it also involves reducing the vehicle speed and sometime waiting for a curb space to become available (see our classification of parking behaviors in Dalla Chiara et al.^11^). Consequently, while cruising for parking distances were still significantly reduced, the magnitude of the reduction was less than the cruising for parking time.

A similar trend is reflected in estimating the impact of providing curb availability information on total drivers’ route times and distances. Moreover, an informed driver might be willing to choose a curb space that is not necessarily closer to the delivery destination(s), compared to an un-informed driver. Therefore, while the cruising for parking times and route times were reduced, the total route distances did not always improve when drivers were provided with curb availability information.

From the perspective of delivery carriers, these time savings are considerable, and by improving drivers’ efficiency the system could support more demand at lower delivery costs. From a policy standpoint, providing delivery drivers with parking information, while reducing their time spent driving, would not directly contribute to reducing vehicle miles traveled and therefore would not directly reduce vehicle emissions. However, reducing cruising for parking could indirectly benefit the environment by improving traffic throughput and potentially reducing the number of vehicles needed to satisfy urban delivery demand. Furthermore, by reducing drivers’ workload and decision-making fatigue, the provision of curb availability information could also improve safety and reduce conflicts between delivery vehicles and other road and curb users.

This paper is the first to explore the impact of providing curb availability information on delivery drivers’ cruising for parking behaviors by way of a real-world pilot of a parking information system in Seattle. The authors hope that this study will encourage more cities to pilot test and explore connected vehicle infrastructure systems for delivery vehicles. While we showed the value of providing curb availability information to delivery drivers, more work should be done to understand the best way to use that information. While drivers had direct access to curb availability information through the OpenPark app, carriers might consider integrating such information in their routing systems to further reduce cruising for parking and improve operational efficiencies.
